# Evaluation of the Effect of Scalp Nerve Block on Bispectral Index Values During Skull Pinning; Prospective Observational Study

**DOI:** 10.3390/medicina62020252

**Published:** 2026-01-25

**Authors:** Halide Hande Şahinkaya, Gözde Gürsoy Çirkinoğlu, Cafer Ak, Sermin Altunbaş, Zeki Tuncel Tekgül

**Affiliations:** 1 Department of Anesthesiology and Reanimation, Izmir City Hospital, 35540 Izmir, Turkey; dr.gozde.gursoy@gmail.com (G.G.Ç.); drsermin@hotmail.com (S.A.); 2 Department of Neurosurgery, Izmir City Hospital, 35540 Izmir, Turkey; slayerkarwyn@gmail.com; 3 Department of Anesthesiology and Reanimation, University of Health Sciences Izmir University Faculty of Medicine, 35540 Izmir, Turkey; zekittekgul@yahoo.com

**Keywords:** bispectral index, craniotomy, local anesthetic, scalp block, skull pinning

## Abstract

***Background and Objectives*:** Scalp nerve block (SNB) is hypothesized to attenuate the physiological response to skull pinning more effectively than local anesthetic (LA) infiltration. This study aimed to compare the two techniques using Bispectral index (BIS) as a primary surrogate measure of cortical arousal. ***Materials and Methods*:** In this prospective observational study, patients undergoing elective craniotomy received either bilateral SNB (Group S, n = 53) or LA infiltration (Group LA, n = 35) based on anesthesiologist preference. Depth of anesthesia was monitored via BIS. The primary outcome was the change in BIS after skull pin insertion. A ΔBIS > 20% from baseline triggered rescue medication (remifentanil/propofol). Secondary outcomes included hemodynamic parameters and rescue requirements. ***Results*:** There was a significant main effect of time on BIS values (*p* < 0.001), indicating that BIS values changed significantly across measurement points. Post-hoc examination of parameter estimates revealed that the Group LA showed significantly greater increases in BIS values compared to the Group S at T1 (*p* = 0.030) and T3 (*p* = 0.024). No significant between-group differences in BIS changes were observed at T5, T10, or T15 time points (*p* > 0.05). Hemodynamic responses (mean arterial pressure and heart rate) were also transiently but significantly higher in Group LA at these time points (*p* < 0.001). The most clinically notable finding was that significantly more patients in Group LA required rescue medication (*p* < 0.001), indicating a greater frequency of clinically significant physiological trespass. ***Conclusions*:** Compared to LA infiltration, SNB was associated with statistically significant reductions in immediate BIS and hemodynamic responses to skull pinning. The key potential clinical implication is the corresponding reduction in the need for rescue anesthetic intervention. These findings support SNB as a technique for enhancing physiological stability, though the direct impact on patient-centered outcomes requires further study. BIS may serve as a useful adjunctive indicator of the cortical response to noxious stimuli.

## 1. Introduction

Maintaining arterial blood pressure within normal limits and controlling the depth of anesthesia in patients undergoing craniotomy are the basis of neuroanesthetic management. Sudden hemodynamic changes due to inadequate analgesia and depth of anesthesia result in increased intracranial pressure [1]. As a result of increased intracranial pressure, bleeding and herniation may occur in a brain with reduced compliance due to intracranial mass.

One of the most painful stage of craniotomy is skull pinning as it triggers noxious stimuli that leads to an increase in heart rate (HR) and mean arterial pressure (MAP), thereby raising cerebral blood flow (CBF) and intracranial pressure (ICP). This situation is undesirable, especially in patient groups where hypertension is contraindicated. Local anesthetic (LA) infiltration can be applied to the pin sites, or a scalp nerve block (SNB) can be performed to block the sensory innervation of the skin of the skull [2,3]. SNB is considered to decrease perioperative cardiovascular complications and enhance recovery after craniotomy [4].

The Bispectral Index (BIS), a processed electroencephalography (EEG) parameter, is a validated monitor of hypnotic depth. EEG waves are analyzed using a sensor placed on the patient’s forehead and converted into numerical data by a processor [5]. In general anesthesia applications where the BIS value is below 60, the incidence of anesthesia awareness decreases [6]. BIS values between 40 and 60 are considered to be an adequate level of anesthesia. It has also been shown to exhibit transient increases in response to intense noxious stimuli, reflecting cortical arousal, even under adequate hypnotic levels [7,8,9,10]. Previous studies have shown that LA infiltration has positive effects on BIS values as there is a relationship between BIS and noxious stimuli [11,12].

This study was designed as a proof-of-concept physiological investigation, not an outcomes trial. The primary findings are to be interpreted as exploratory, generating hypotheses for future randomized trials. The primary aim is to evaluate the BIS value changes during skull pinning under SNB. Secondary aim is to evaluate the hemodynamic parameters. Our hypothesis is that SNB causes less change in BIS values compared to LA infiltration at the site of skull pins. Therefore, we hypothesized that BIS could serve as a sensitive surrogate marker to compare the efficacy of two regional techniques in blunting the central nervous system’s response to the discrete noxious stimulus of skull pinning.

## 2. Materials and Methods

### 2.1. Ethical Approval

This prospective observational study was conducted at Izmir City Hospital between 25 May and 20 August 2025. Ethical approval was obtained from the local ethics commitee (No. 2024/217, Approval date: 4 December 2024), and the study was registered at ClinicalTrials.gov (NCT06984653-21 May 2025). Ethical standards in the Declaration of Helsinki were considered. Patients were informed about the study and their written consent was obtained.

### 2.2. Study Population

Patients (≥18 years) undergoing elective craniotomy for supratentorial mass excision with skull pin fixation were prospectively enrolled. Patients who met eligibility criteria were classified as American Society of Anesthesiologists (ASA) physical status I-III.

Patients with uncontrolled hypertension, hypersensitivity to local anesthetics, treatment that can affect hemodynamic parameters, who refused to involve in the study and emergency surgery were excluded from the study.

### 2.3. Study Protocol

This was a prospective observational study in which standard clinical care was not altered. Anesthetic management and surgical procedures were performed according to routine institutional practice. Data collection was conducted by an anesthesiologist who was not involved in the study design or outcome assessment.

Demographic variables, including age, gender, body weight, comorbidities, and ASA physical status, were recorded.

Standard monitoring included electrocardiography, pulse oximetry, invasive arterial blood pressure monitoring, and BIS monitoring. To optimize EEG signal quality, the skin on the forehead was cleansed with alcohol before placement of the BIS sensor (Bispectral Index™ Monitoring System, Covidien, Minneapolis, MN, USA).

After preoxygenation, anesthesia was induced with propofol (2 mg/kg), remifentanil (1 µg/kg), and rocuronium bromide (0.6 mg/kg). Endotracheal intubation was performed following neuromuscular blockade. Anesthesia was maintained with continuous infusions of propofol and remifentanil, targeting a BIS value between 40 and 50. Intraarterial blood pressure was also monitored.

### 2.4. Allocation Procedure

The allocation of patients to either the scalp nerve block (Group S) or local infiltration (Group LA) group was determined solely by the routine clinical preference of the attending anesthesiologist on the day of surgery. A total of 3 attending anesthesiologists participated in patient management during the study period. During the study period, each participating anesthesiologist consistently used only one of the two techniques as their personal routine practice; that is, anesthesiologists did not switch between techniques based on individual patient or case characteristics. Patients were enrolled consecutively, and no selective enrollment based on anticipated response or surgeon/anesthesiologist preference occurred.

In Group S, a bilateral scalp block targeting the supraorbital, supratrochlear, zygomaticotemporal, auriculotemporal, greater occipital, and lesser occipital nerves was performed using a total of 25 mL of 0.25% bupivacaine.In Group LA, local infiltrative anesthesia was administered with 5 mL of 0.25% bupivacaine at each skull pin insertion site.

After skull-pin insertion, a difference of more than 20% from the baseline values was regarded as clinically significant and was treated by iv bolus dose of 1 mcg/kg remifentanil and 0.5 mg/kg propofol as rescue drugs to prevent increased hemodynamic response. It is acknowledged that this rescue intervention, by design, directly affects the very parameters (BIS and hemodynamics) that are the outcomes of the study. Therefore, measurements taken after a rescue intervention were included in the analysis but their interpretation is made with this confounding influence in mind.

The primary outcome was the change in BIS values from baseline at 1 min (T1), 3 min (T3), 5 min (T5), 10 min (T10) and 15 min (T15) after skull pin insertion.

Secondary outcomes included intraoperative hemodynamic parameters (systolic, diastolic, and mean arterial pressure (MAP), and heart rate (HR)), rescue anesthetic requirements during skull pinning, and total intraoperative propofol and remifentanil consumption.

### 2.5. Statistical Analysis

This was an exploratory observational study. A formal a priori sample size calculation was not performed; the sample size was based on a convenience sample of eligible patients available during the study period. Therefore, the study was not prospectively powered to detect a predefined effect size.

*Primary Analysis—Repeated Measures:* The primary outcome, BIS changes across the five time points (1, 3, 5, 10, and 15 min), was analyzed using a linear mixed-effects model. This model accounts for the within-subject correlation of repeated measurements. The model included fixed effects for Group (S vs. LA), Time (as a categorical factor), and the crucial Group × Time interaction, with a random intercept for each subject. The significance of the Group × Time interaction term was the primary test of interest. If this interaction was significant, it would indicate that the pattern of BIS change over time differed between groups.

*Secondary and Baseline Comparisons:* Continuous baseline and secondary outcome variables were tested for normality using the Shapiro-Wilk test. Variables with a normal distribution are presented as mean ± standard deviation (SD) and compared using independent *t*-tests. Variables with a skewed distribution are presented as median [interquartile range (IQR)] and compared using the Mann-Whitney U test. Categorical data are presented as number (%) and compared using the Chi-square or Fisher’s exact test, as appropriate.

To address potential confounding due to the non-randomized design, a secondary multivariable linear regression analysis was performed for the BIS value at the most significant time point (T3). The model adjusted for age, ASA status (dichotomized as I/II vs. III), and the presence of COPD. Results are reported as an adjusted mean difference with a 95% confidence interval (CI).

A two-tailed *p*-value < 0.05 was considered statistically significant for the overall mixed-model interaction and main effects. For post-hoc comparisons, the adjusted significance threshold (Sidak) was reported. Analyses were performed using SPSS software (version 30.0, IBM Corp., Chicago, IL, USA) with the linear mixed-effects module.

## 3. Results

**Figure 1:**
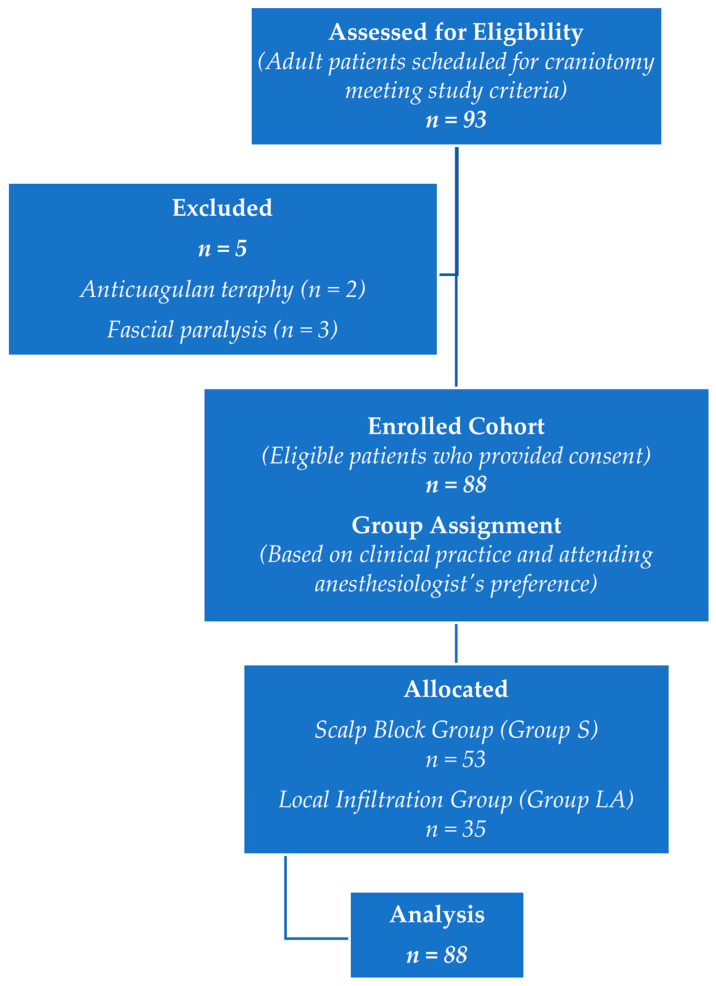
Flow chart.

A total of 93 patients met eligibility criteria for the study. Two of these patients were excluded from the study because they were receiving anticoagulant therapy and three had facial paralysis, making scalp block inappropriate. Consequently, a total of 88 patients were included in the study and the results were analysed, with 53 patients in the scalp block group and 35 patients in the local anaesthetic infiltration group (Figure 1).

Demographic data of the patients are given in Table 1.

The distribution of chronic obstructive pulmonary disease (COPD) differed significantly between the groups (*p* = 0.009), indicating a potential confounding variable. In the multivariable linear regression model, the scalp block group continued to demonstrate a significantly lower BIS value at T3 compared to the local infiltration group (adjusted mean difference: −1.85 points; 95% CI: −3.1 to −0.6; *p* = 0.004) (Table 2). The presence of COPD was also independently associated with a higher BIS value at T3 (adjusted B: 2.20; 95% CI: 0.6 to 3.8; *p* = 0.009).

The linear mixed-effects model was used to analyze BIS values over time between the scalp block and local infiltration anesthesia groups (Table 3). The model included group, time, and their interaction as fixed effects. The overall model showed excellent fit (conditional R^2^= 0.779). There was a significant main effect of time on BIS values (F[5, 430] = 43.81, *p* < 0.001, η^2^p = 0.337), indicating that BIS values changed significantly across measurement points. No significant main effect of group was found (F[1, 86] = 1.41, *p* = 0.239), suggesting no overall difference in BIS values between the two groups. However, a significant group × time interaction was detected (F[5, 430] = 3.71, *p* = 0.003, η^2^p = 0.041). Post-hoc examination of parameter estimates revealed that the Group LA showed significantly greater increases in BIS values compared to the Group S at T1 (1.36 points greater increase, *p* = 0.030) and T3 (1.42 points greater increase, *p* = 0.024). No significant between-group differences in BIS changes were observed at T5, T10, or T15 time points (all *p* > 0.05).

**Figure 2:**
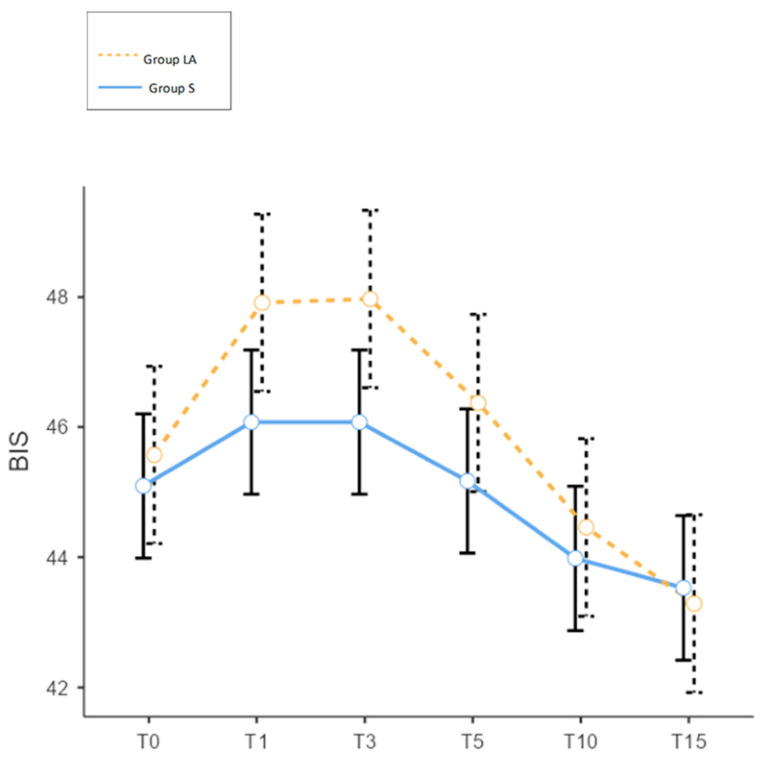
BIS changes during skull pinning between the groups, Group S: Scalp nerve block group, Group LA: Local infiltrative anesthesia group, T0: Before skull pinning, T1: 1 min after skull pinning, T3: 3 min after skull pinning, T5: 5 min after skull pinning, T10: 10 min after skull pinning, T15: 15 min after skull pinning.

Estimated marginal means showed that BIS values peaked at T1 and T3 (47.0 for both) before declining to 43.4 by T15 in both groups, though the magnitude of early increase was greater in the Group LA (Figure 2). Model assumptions were met as confirmed by examination of Q-Q plots and residual diagnostics.

Hemodynamic parameters are given in Table 4 and Table 5. There was an increase in HR during the first and third minute of skull pinning in the groups when compared to baseline measurements. The increase was higher in Group LA and this found to be statistically significant (*p* = 0.001). The MBP were measured higher at 1st and 3rd minute of skull pinning. Patients in Group LA had higher measurements when compared with the patients in Group S. The difference between the groups was statistically significant (*p* < 0.001).

During skull pinning, 2 patients in Group S and 14 patients in Group LA received additional intravenous remifentanil bolus, while 4 patients in Group S and 15 patients in Group LA received additional intravenous propofol bolus. A statistically significant difference was found between the groups in terms of additional drug administration (*p* < 0.001). However, no significant difference was observed between groups in terms of total propofol and remifentanil consumption (*p* = 0.142 and *p* = 0.094, respectively).

## 4. Discussion

The present prospective observational study demonstrates that bilateral scalp nerve block (SNB) was associated with significantly smaller changes in Bispectral Index (BIS) values and greater hemodynamic stability during skull pin insertion compared to local anesthetic (LA) infiltration. Furthermore, patients receiving SNB required fewer rescue doses of anesthetic agents during this noxious phase.

While the analgesic efficacy of scalp blocks in reducing postoperative pain and opioid consumption is well-documented in the literature [13,14], and their superiority over local infiltration in blunting the hemodynamic and sympathetic stress response to skull pinning is recognized [15,16,17], a critical aspect has remained less explored. Previous investigations have primarily relied on indirect physiological surrogates of stress, such as heart rate, blood pressure, or plasma catecholamine levels [18], or have focused on postoperative outcomes. The cortical electrophysiological response—the direct central nervous system reaction to the noxious stimulus—has not been similarly quantified and compared between these techniques using a real-time, objective monitor.

This study addresses this gap by introducing continuous Bispectral Index (BIS) monitoring as a primary outcome measure. The BIS algorithm, derived from the frontal electroencephalogram (EEG), provides a processed numerical index of cortical activity and hypnotic depth. Crucially, it has been validated as a sensitive tool that exhibits transient increases in response to intense noxious stimuli under general anesthesia, reflecting cortical arousal even when hemodynamic changes are attenuated [9]. Therefore, BIS serves not as a measure of pain per se, but as an objective surrogate marker of the integrated cortical component of the nociceptive-stress response.

The novel contribution of our work lies in leveraging this modality to directly quantify and compare the magnitude of cortical arousal elicited by skull pin fixation under SNB versus LA infiltration. We provide high-resolution, second-by-second data demonstrating that a comprehensive scalp block results in a significantly more attenuated BIS trajectory in the immediate minutes following the stimulus. This finding adds a new, neurophysiological dimension to the understanding of SNB’s mechanism, suggesting its benefits encompass a more profound central modulation of the stress cascade, complementing its known peripheral analgesic and hemodynamic effects.

Both SNB and LA infiltration are established methods to attenuate the sympathetic response to pinning. However, SNB, by providing more comprehensive multi-nerve blockade, has been shown superior in blunting hemodynamic surges and stress hormone release [18,19]. Our findings align with this, showing SNB’s stabilizing effect on blood pressure and heart rate. The transient nature of the pinning stimulus likely explains why these hemodynamic differences resolved by the 10- and 15-min marks. More importantly, our study extends beyond hemodynamics by providing second-by-second neurophysiological data. The significantly attenuated ΔBIS in the SNB group during the first three minutes indicates a more effective dampening of the integrated cortical response to noxious input. This suggests SNB’s superiority may extend beyond peripheral somatic analgesia to a more profound modulation of central nervous system processing.

### 4.1. Interpreting the BIS Signal: Surrogate Value and Limitations

The interpretation of our BIS data requires caution. BIS is a validated monitor of hypnotic depth but is not a specific monitor of analgesia [20]. It is influenced by frontal electromyographic (EMG) activity, which can itself increase with noxious stimuli [21,22]. Therefore, the smaller ΔBIS observed with SNB likely reflects a more effective blunting of the integrated cortical and subcortical (including myogenic) response. In this context, BIS should be viewed as a physiologically plausible surrogate marker of nociceptive-arousal, not a direct measure of pain. Our protocol excluded patients with BIS < 40 to mitigate the limited BIS response under deep sedation [19], and the observed changes are consistent with BIS increases documented during nociceptive procedures in other settings [23,24,25].

### 4.2. Clinical Relevance: Between Statistical Significance and Patient Benefit

The clinical meaningfulness of a 1–2 point BIS difference, while statistically significant, warrants careful consideration. The literature lacks a consensus on the minimal clinically important difference (MCID) for BIS in this specific context. Consequently, our BIS findings should be viewed as indicative of a favorable physiological signal—a more stable neurophysiological milieu—rather than definitive proof of superior clinical analgesia.The more direct clinical implication comes from the significantly reduced need for rescue anesthetic medication in the SNB group. This aligns with literature suggesting scalp blocks improve hemodynamic stability and logically complements studies showing SNB reduces postoperative pain and opioid consumption [26,27]. Reduced intraoperative anesthetic requirements may also contribute to faster emergence and extubation [28]. However, we must acknowledge that the rescue protocol itself creates a methodological confound: the administration of rescue drugs in the LA group actively lowered BIS, likely attenuating the true inter-group difference at later time points (5, 10, 15 min). Therefore, the most reliable interpretation of our BIS data is confined to the immediate, pre-rescue response (ΔBIS1, ΔBIS3).

The observed peak hemodynamic differences (~14 mmHg MAP, ~10 bpm HR), though significant, were transient, and both groups remained within clinically acceptable ranges. Their primary relevance is preventive and probabilistic: SNB reduced the likelihood of requiring pharmacological intervention to maintain targets, a valuable attribute in neuroanesthesia where tight physiological control is paramount.

### 4.3. Limitations

The most significant limitation is the non-randomized, observational design with allocation based on anesthesiologist preference. This introduces potential for confounding. We addressed this by performing a multivariable linear regression adjusting for key covariates, including the significant baseline imbalance in COPD prevalence. This adjusted analysis confirmed that SNB remained independently associated with a lower ΔBIS3 strengthening the inference that the effect is not merely an artifact of uneven comorbidity distribution. Nonetheless, residual confounding (e.g., by unmeasured anesthesiologist or surgical factors) cannot be excluded, and our findings must be interpreted as demonstrating a robust association, not causation. Furthermore, due to its focused physiological design, the study did not collect data on several relevant clinical outcomes: vasopressor use, surgeon-rated surgical conditions, postoperative pain scores beyond the immediate period, time to extubation, or long-term recovery metrics. This limits our ability to directly link the observed physiological advantages to tangible patient-centered benefits as reducing POCD, shortening eye-opening time, orientation force recovery time, extubation time, PACU stay duration, and decreasing anesthesia drug dosage [29]. Finally, we did not measure direct markers of sympathetic activity (e.g., plasma catecholamines, heart rate variability) as well.

### 4.4. Conclusions and Future Direction

In conclusion, bilateral SNB was associated with superior attenuation of the cortical (BIS) and hemodynamic response to skull pinning, alongside a reduced need for rescue anesthesia. While the absolute BIS differences were modest, they represent a consistent, biologically plausible signal of enhanced physiological stability. The primary contribution of this study is to provide objective neurophysiological evidence that complements the existing clinical outcome literature, thereby strengthening the rationale for SNB. These findings are hypothesis-generating. The definitive assessment of clinical superiority requires future randomized controlled trials powered to detect differences in patient-centered outcomes such as recovery quality, pain scores, and opioid consumption, building upon the physiological framework established here.

## Figures and Tables

**Table 1:** Baseline and demographic characteristics of the study groups.

	Group S (n = 53)	Group LA (n = 35)	*p*-Value
Age (y)	55.7 ± 13.9	49.6 ± 16.8	0.067
Gender (F/M)	28 (52.8%)/25 (47.2%)	15 (42.8%)/20 (57.2%)	0.360
HT	27 (50.9%)	15 (42.8%)	0.457
DM	9 (16.9%)	6 (17.1%)	0.984
CAD	9 (16.9%)	5 (14.3%)	0.735
COPD	15 (28.3%)	2 (5.7%)	0.009 *
Smoking	27 (50.9%)	12 (34.2%)	0.124
ASA I II III	1 (1.88%) 29 (54.7%) 23 (43.4%)	4 (11.4%) 12 (34.2%) 19 (54.3%)	0.055

Data are given as mean ± standart deviation (SD) or numbers (%). * *p* < 0.05 is considered statistically significant. Group S: Scalp nerve block group, Group LA: Local infiltrative anesthesia group, y: year, F: Female, M: Male, HT: Hypertension, DM: Diabetes Mellitus, CAD: Coronary artery disease, COPD: Chronic obstructive pulmonary disease, ASA: American Society of Anesthesiologists Physical Condition Classification.

**Table 2:** Results of the multivariable linear regression analysis for BIS value changes at 3 min Post Skull-Pin Insertion.

Variable	Adjusted Mean Difference	95% Confidence Interval	*p*-Value
Group (S vs. LA)	1.85	0.6–3.1	0.004 *
Age (years)	0.05	−0.01–0.11	0.099
ASA Status (III vs. I/II)	0.90	−0.4–2.2	0.171
COPD (Yes vs. No)	2.20	0.6–3.8	0.009 *

Values are presented as adjusted mean difference (95% confidence interval), * *p* < 0.05 is considered statistically significant, Group S: Scalp nerve block group, Group LA: Local infiltrative anesthesia group, ASA: American Society of Anesthesiologists, COPD: Chronic Obstructive Pulmonary Disease.

**Table 3:** Fixed Effects Results from Linear Mixed Model Analysis of BIS Values.

Effect	F	df1	df2	*p*-Value	Partial η^2^	95% CI for η^2^
Intercept	114.65	1	86.0	<0.001 *	—	—
Group (S vs. LA)	1.41	1	86.0	0.239	0.016	[0.000, 0.089]
Time (Measurement Point)	43.81	5	430.0	<0.001 *	0.337	[0.272, 0.386]
Group × Time Interaction	3.71	5	430.0	0.003 *	0.041	[0.010, 0.067]

Effect size was expressed as partial eta-squared (η^2^p), * *p* < 0.05 was considered as statistically significant, Group S: Scalp nerve block group, Group LA: Local infiltrative anesthesia group, F = F-statistic representing the ratio of variance explained by the effect to unexplained variance, df1 = numerator degrees of freedom (effect df), df2 = denominator degrees of freedom (error df), CI = Confidence Interval. 95% CIs indicate the range within which the true population mean is expected to fall with 95% confidence, based on the sample data.

**Table 4:** Intraoperative heart rate before and after skull pin insertion.

	Group S	Group LA	*p*-Value
Baseline HR	63.7 ± 11.5	67.3 ± 13.2	0.174
HR, 1 min	66.6 ± 12.8	76.3 ± 13.1	0.001 *
HR, 3 min	66.3 ± 12.5	75.6 ± 12.9	0.001 *
HR, 5 min	63.6 ± 12.4	71.5 ± 12.9	0.005 *
HR, 10 min	62.8 ± 11.3	67.7 ± 13.4	0.069
HR, 15 min	63.4 ± 11.4	66.4 ± 12.4	0.250

Data were given as mean ± standart deviation (SD), * *p* < 0.05 was considered as statistically significant, HR: Heart rate (beats per minute), min: Minute, Group S: Scalp nerve block group, Group LA: Local infiltrative anesthesia group.

**Table 5:** Intraoperative mean arterial pressure before and after skull pin insertion.

	Group S	Group LA	*p*-Value
Baseline MAP (mmHg)	83.9 ± 12.8	84.8 ± 10.6	0.719
MBP1	92.2 ± 13.9	106.5 ± 16.2	<0.001 *
MBP3	90 ± 13.6	105.1 ± 21.4	<0.001 *
MBP5	86.4 ± 11.1	93.6 ± 10.6	0.003 *
MBP10	82.9 ± 10.5	85.5 ± 9.1	0.235
MBP15	81.7 ± 10.4	80.9 ± 9.9	0.724

Data were given as mean ± standart deviation (SD), * *p* < 0.05 was considered as statistically significant, MAP: Mean arterial pressure (mmHg), Group S: Scalp nerve block group, Group LA: Local infiltrative anesthesia group.

## Data Availability

The original contributions presented in this study are included in the article. Further inquiries can be directed to the corresponding author.

## Abbreviations

The following abbreviations are used in this manuscript:

BIS	Bispectral index
SNB	Scalp nerve block
LA	Local anesthetic
EEG	Electroencephalography
MAP	Mean arterial pressure
HR	Heart rate
CBF	Cerebral blood flow
ICP	Intracranial pressure
EMG	Electromyography
